# *Cheilomenes sexmaculata* (Coccinellidae: Coleoptera) as a potential biocontrol agent for aphids based on age-stage, two-sex life table

**DOI:** 10.1371/journal.pone.0228367

**Published:** 2020-09-25

**Authors:** Khalid Abbas, Muhammad Shah Zaib, Muhammad Zakria, Umm-e Hani, Syed Muhammad Zaka, Muhammad Noor-ul Ane

**Affiliations:** 1 Department of Entomology, Faculty of Agricultural Sciences and Technology, Bahauddin Zakariya University, Multan, Pakistan; 2 Agricultural science & technology research institute, ANU, Andong, Korea; University of Saskatchewan College of Agriculture and Bioresources, CANADA

## Abstract

The Zigzag ladybird beetle, *Cheilomenes sexmaculata* (Fabricius) (Coleoptera: Coccinellidae), is a biological control agent that feeds on a variety of aphid species. Life table and predation data of *C*. *sexmaculata* were collected under laboratory conditions at 25±2⁰C, 60±5% RH and L14: D10 h in connection with feeding on four different aphid species; *Lipaphis erysimi* (Kaltenbach), *Myzus persicae* (Sulzer), *Aphis nerii* (Boyer de Fonscolombe) and *Diuraphis noxia* (Mordvilko). Larval development of *C*. *sexmaculata* was long when fed on *M*. *persicae* (12.18 days) and shorter on *D*. *noxia* (10.64 days). The male’s lifespan was longer on *M*. *persicae* (26.70 days) and shorter on *L*. *erysimi* (23.67 days). Fecundity was maximum when the beetle was fed *D*. *noxia* (316.8 eggs/female) and minimum on *M*. *persicae* (199.1 eggs/female). Net reproductive rate, intrinsic rate of increase and finite rate of increase were highest on *D*. *noxia* with values of 158.4 (offspring individual^-1^), 0.22 d^-1^, and 1.24 d^-1^, respectively whereas the respective parameters were lowest on *L*. *erysimi* (99.5 offspring individual^-1^, 0.19 d^-1^, and 1.20 d^-1^, respectively). However, the mean of the generation (*T*) was shorter on *A*. *nerii* (22.48 d^-1^) and longer on *M*. *persicae* (24.68 d^-1^). Based on life table parameters obtained under laboratory conditions, the most appropriate host of *C*. *sexmaculata* was *D*. *noxia*. This study should help us to improve mass rearing and use of *C*. *sexmaculata* in the biological control of aphids on field and horticultural crops.

## Introduction

Aphids (Hemiptera: Aphididae) are important insect pests of various cultivated plants [1]. They suck the cell sap of plants, weaken the latter and act as vectors of various virus-induced diseases [2]. Aphids can rapidly build up their population and their honeydew secretions result in sooty mold on the plants. They can change their host’s metabolism by disturbing their host’s hormonal balance. Aphid attacks may lead to a plant’s early death or will reduce the yield of crops at later stage [3]. *Aphis nerii*, Kaltenbach (oleander aphid), *Myzus persicae*, Sulzer (green peach aphid), *Diuraphis noxia*, Kurdjumov (Russian wheat aphid), and *Lipaphis erysimi*, Kaltenbach (mustard aphid) are among the most important pests of cultivated and ornamental plants [4]. *Lipaphis erysimi*, is most damaging towards Brassicaceae plants: mustard, rape, cabbage, cauliflower, broccoli, and radish [5]. *Myzus persicae* is a cosmopolitan pest that feeds on more than 50 plant families [5], including agro-industrial crops and horticultural crops [6].

With a host range of more than 140 species of Poaceae plants, *Diuraphis noxia* attacks cereal crops worldwide [7]. It injects a toxin into plants during feeding which causes rolling and white streaking of plant leaves. Crop yield loss of wheat under heavy attack by *D*. *noxia* can be as high as 80–100% [8]. *Aphis nerii* feeds on plants of the families of Apocynaceae and Asclepiadaceae [9] and has also been reported on wheat and brassica in Pakistan [10]. *Aphis nerii* reproduces by obligate parthenogenesis and sequester those toxic chemicals (cardenolides), which act as a defensive mechanism against its natural enemies [11].

Excessive use of pesticides causes environmental pollution, human health issues and resistance problems in aphids [12, 13]. These factors make it important that we explore alternative control methods (e.g. biological control) that should be environment-friendly and risk-free for human health.

Natural enemies (predators, parasitoids, and entomopathogens) have been used to control aphid populations [12]. Ladybirds (Coleoptera: Coccinellidae) are potent predators of various small herbivorous insects such as aphids [13]. The ladybird beetle, *C*. *sexmaculata* (Fab.), is widely distributed in Pakistan, India, and other south Asian countries [14]. This beetle is a generalist predator of soft-bodied insects i.e. aphids, whitefly, thrips, scale insects, mealy bugs [15]. However, only very few studies focused on the biological aspects of *C*. *sexmaculata*. There is therefore a need for detailed observations on the survival and reproduction of *C*. *sexmaculata* when reared on different aphid species. For mass rearing ventures and use of *C*. *sexmaculata* in the biological control of pests, it is important to know certain demographic aspects including stage differentiation and predation rate of predators [16, 17]. The age-stage two-sex life table provides a better understanding of biological aspects including stage differentiation than traditional life tables [17]. The present study, therefore, uses the age-stage two-sex life table for a more complete understanding of *C*. *Sexmaculata*’s biological role against different aphid species. This study should help us improve mass rearing and the use of *C*. *sexmaculata* in the biological control of aphids.

## Material and methods

### Aphids rearing

In 2019, four aphid species (*A*. *nerii*, *M*. *persicae*, *D*. *noxia* and *L*. *erysimi*) were collected from their host plants from the agricultural fields (latitude 30°15'29.9"N, longitude 71°30'54.6"E) of the Faculty of Agricultural Sciences and Technology, Bahauddin Zakariya University, Multan Pakistan and were reared on their respective host plants *Nerium oleander*, *Prunus persica*, *Triticum aestivum* and *Brassica campestris*. The aphids were maintained in the laboratory in plastic cages (51 **×** 45 cm) along with their respective hosts under condition (25 ± 2°C and 70 ± 5% RH with a photoperiod of 14L:10D h) [18], and then they were used for the studies with *C*. *sexmaculata*.

### *Cheilomenes sexmaculata* rearing

Larvae of *C*. *sexmaculata* were collected in the early morning from *Calotropis procera* (Aiton) (Gentianales: Asclepiadaceae) located at the head Muhammad Wala fields of Multan (latitude: 30°11′54.97N, longitude: 71°28′7.33E), Punjab, Pakistan at the start of February 2019. The culture was maintained in an incubator (25±1˚C and 60±2% R.H.) with a photoperiod of 14L:10D h [19]. The collected larvae were individualized in petri dishes (6cm diameter). Different aphid species, i.e. *A*. *nerii*, *M*. *persicae*, *D*. *noxia*, and *L*. *erysimi* were supplied as food to the larvae. Emerging adults were reared in plastic cages (14 × 8 × 10 cm) with different aphid species. Corrugated filter papers were used as an oviposition substrate for the beetles in the rearing jars. Collected eggs from their adult females were placed in 10-cm Petri dishes containing moist filter paper at the bottom to obtain larvae. Mature and immature stages of *C*. *sexmaculata* were provided with aphids as their food [20].

### Life table studies

Twenty fresh eggs of *C*. *sexmaculata* were taken from the third-generation population of their respective hosts and kept separately in Petri dishes (6cm diameter) in an incubator with same conditions used for *C*. *sexmaculata* rearing. Egg development and survival was recorded daily. After egg hatching, first instars of *C*. *sexmaculata* were shifted in petri dishes (6cm diameter) individually. Twenty to thirty similar sized aphids (nymphs and adults) from lab aphids culture were provided to *C*. *sexmaculata* larvae and leaves of respective aphid’s host plant were placed on moist filter paper on the bottom of Petri dishes. Consumed aphids were recorded [21] and unconsumed aphids were removed after every 24h. Pupae were kept on the moist filter paper in same Petri dishes of their respective larvae. The development and survival of all immature stages of *C*. *sexmaculata* were recorded on a daily bases [18, 19, 22]. Newly emerged adults were paired and introduced into a plastic jar (9 × 6 cm) containing 100 to 200 aphids. Water-soaked cotton was provided to the adults and a piece of corrugated filter paper was added as an oviposition substrate. The adult’s survival, egg laying, and aphids consumption were recorded daily [22, 23].

### Statistical analysis

The life-history traits (development and reproductive parameters) and aphid consumption of *C*. *sexmaculata* were analyzed by one-way analysis of variance (ANOVA) and means were compared by using Least significant difference (LSD) test (*P* = 0.05). This analysis was done by using the statistical package SAS [24]. Life table parameters were calculated by using TWO SEX-MS Chart. The raw data were used to calculate the age-stage–specific survival rate (*s*_*xj*,_ where *x* = age in days and *j* = stage), age-stage specific fecundity (*f*_*xj*_), age-specific survival rate (*l*_*x*_), age-specific fecundity (*m*_*x*_), age-specific net maternity (*l*_*x*_*m*_*x*_), age-stage life expectancy(*e*_*xj*_), age-stage reproductive value (*v*_*xj*_), and life table parameters (*R*_0_, net reproductive rate; *r*, intrinsic rate of increase; *λ*, finite rate of increase; and *T*, the mean generation) [25]. In the age-stage, two-sex life table, the age-specific survival rate *l*_*x*_, *m*_*x*,_ and *R*_o_ were calculated as (1 and 2):

$$l_{x}=\sum_{j=1}^{k}S_{xj}$$ (1)

$$m_{x}=\frac{\sum_{j=1}^{k}S_{xj}f_{xj}}{\sum_{j=1}^{k}S_{xj}}$$ (2)

Where *k* is the number of stages. The net reproductive rate *R*_0_ is the mean number of offspring laid by the individual during its entire life span. It was calculated by the following Eq (3):

$$R_{0}=\sum_{x=0}^{\infty }l_{x}m_{x}$$ (3)

The intrinsic rate of increase (*r*) was estimated using the iterative bisection method and corrected with the Euler–Lotka Eq (4) with the age indexed from 0 [26]:

$$\sum_{x=0}^{\infty }e^{-r(x+l)}l_{x}m_{x}=1$$ (4)

The finite rate (*λ*) was calculated as (5):

$$\lambda =e^{r}$$ (5)

The mean generation time is defined as the length of time that a population needs to increase to *R*_0_-fold of its population size at the stable age-stage distribution, and is calculated as (6):

$$T=In\;R_{0}/r$$ (6)

The life expectancy (*e*_*xj*_) is the length of time that an individual of age *x* and stage *j* is expected to live and it is calculated Eq (7) according to as [17].

$$e_{xj}=\sum_{i=x}^{\infty }\sum_{y=j}^{\beta }{s\prime }_{iy}$$ (7)

## Results

Development of first and fourth instars of the zigzag beetle only varied significantly (*P*<0.05) on different aphids (Table 1). The first instar completed its development in 1.00 days on *D*. *noxia*, which was significantly faster (*F* = 2.17; F_3,76,_ and *P*<0.0001) than 1.45, 2.05 and 2.25 days on *L*. *erysimi*, *M*. *persicae A*. *nerii*, respectively. Fourth instars of the *C*. *sexmaculata* completed their quick development in 2.10 days while feeding on *A*. *nerii* and shared the same statistical rank on *M*. *persicae* (2.30 days) and *D*. *noxia* (2.40 days).

**Table 1:** Development and reproductive parameters (mean ± SE) of *C*. *sexmaculata* on different aphid species.

Developmental Stage	Aphid species				Statistical parameters		
	*Aphis nerii*	*Myzus persicae*	*Diuraphis noxia*	*Lipaphis erysimi*	*F*.*value*	*df*, Edf*	*P value*
Eggs (days)	4.00a±0.20	3.60a±0.17	3.30a±0.16	3.60a±0.12	2.17	3,76	0.0985
First instar (days)	2.25a±0.12	2.05a±0.18	1.00b±2.19	1.45b±0.18	11.1	3,76	<0.001
Second instar (days)	1.11a±0.10	1.00a±0.00	1.10a±1.07	1.30a±0.13	1.06	3,70	0.3719
Third instar (days)	1.11a±0.00	1.05a±0.05	1.47a±1.12	1.20a±0.09	0.96	3,70	0.4158
Fourth instar (days)	2.10b±0.10	2.30b±0.18	2.47ab±0.12	2.80a±0.15	4.72	3,70	0.001
Pupa (days)	3.10a±0.18	3.25a±0.16	2.65a±3.48	3.30a±0.28	2.04	3,70	0.1165
Total duration of Immature stages (egg-adult) (days)	13.67a±0.35	13.26ab±0.23	12.42b±0.35	13.83a±0.43	3.79	3,70	0.01
Male longevity (days)	24.30b± 4.70	26.70a±3.53	26.33a±2.19	23.67c±3.70	0.19	3,35	<0.0001
Female longevity (days)	24.44 ± 1.27	25.90±1.60	28.00±0.83	25.60±0.86	1.56	3,35	0.2172
Pre-oviposition period (days)	07.00a ± 2.50	06.33b±0.88	5.33b±1.33	5.67b±1.45	0.18	3,35	0.0211
Oviposition period (days)	15.70b ± 2.33	18.33a±1.90	14.70b±1.33	10.33c±6.90	0.87	3,35	<0.0001
Post-oviposition period (days)	11.00a ± 3.21	1 1.33a±3.80	7.33c±1.70	9.70b±4.33	0.72	3,35	<0.0001
Fecundity (eggs/female)	260.56ab±24.80	235.80bc±20.30	316.80a±25.07	199.10c±17.10	5.03	3,35	0.003

Note: Mean followed by different letters in the same row are significantly different (*P* = 0.05)

* *df* = Degree of freedom (N-1): *Edf* = Error degree of freedom

Feeding with different aphid species significantly (*F* = 2.15; df = 3 and *P*<0.0001) influenced the total duration of immature stages. The total developmental duration of immature stages was longer on *A*. *nerii* and *L*. *erysimi* (13.67 and 13.50 days, respectively), but when *D*. *noxia* was given, it only lasted 12.37 days. A significant difference in adult longevity of males (*F* = 0.19; F_3,35_ and *P*<0.0001) was observed, but no significant difference was recorded for females longevity (*F* = 1.56; F_3,35_ and *P* = 0.2172) when different aphid species were provided to them as food (Table 1). Males lived longest when fed *M*. *persicae* and *D*. *noxia* (26.7 and 26.33 days, respectively) and shortest when *L*. *erysimi* (23.67 days) was their diet.

When the beetles fed on different aphids as food, significant differences (*F* = 0.18; 3,35 and *P* = 0.0211) regarding pre-oviposition period were recorded (Table 1). The oviposition period also varied significantly (*F* = 0.87; F_3,35_ and *P*<0.0001) when the beetles were fed on different aphid species. The oviposition period was maximum (18.33 days) when *C*. *sexmaculata* fed on *M*. *persicae* and minimum on *L*. *erysimi* (10.33 days).

Fecundity of *C*. *sexmaculata* varied significantly (*F* = 5.03; F_3,35_ and *P*<0.01) on different aphids. Maximum fecundity recorded when *D*. *noxia* (316.8 eggs/female) was given as food and `minimum fecundity was recorded when *M*. *persicae* (199.1 eggs/female) was given as food (Table 1).

Life table parameters: *r*, λ, and *R*o (0.22 d^-1^,1.24 d^-1^ and 158.4 offspring, respectively) were higher on *D*. *noxia* as food than with the other tested aphid species (Table 2). Mean generation time (*T*) was higher (24.68 d^-1^) on *L*. *erysimi* than on *A*. *nerii* (23.494 d^-1^), *M*. *persicae* (24.006 d^-1^) and *D*. *noxia* (23.494 d^-1^). The maximum gross reproductive rate (*GRR*) of the zigzag beetle was 172.2 offspring when fed on *D*. *noxia* followed by 131.92 and 125.67 offspring on *A*. *nerii* and *M*. *persicae*, respectively.

**Table 2:** Life table parameters of *C*. *sexmaculata* on different aphid species calculated by age-stage, two-sex life table.

Parameters	Aphid species			
	*Aphis nerii*	*Myzus persicae*	*Diuraphis noxia*	*Lipaphis erysimi*
*r* (d^-1^)	0.21	0.20	0.22	0.19
*λ* (d^-1^)	1.24	1.22	1.24	1.20
*R*_*o*_ (Offspring individual^-1^)	117.25	117.9	158.4	99.55
*T* (d)	22.48	24.0	23.49	24.68
*GRR*(Offspring)	131.92	125.7	172.2	115.0

Note: *r* = intrinsic rate of increase, *λ* = finite rate of increase, *R*_*o* =_ net reproductive rate *T* = the mean of generation, *GRR* = the gross reproductive rate

**Fig 1:**
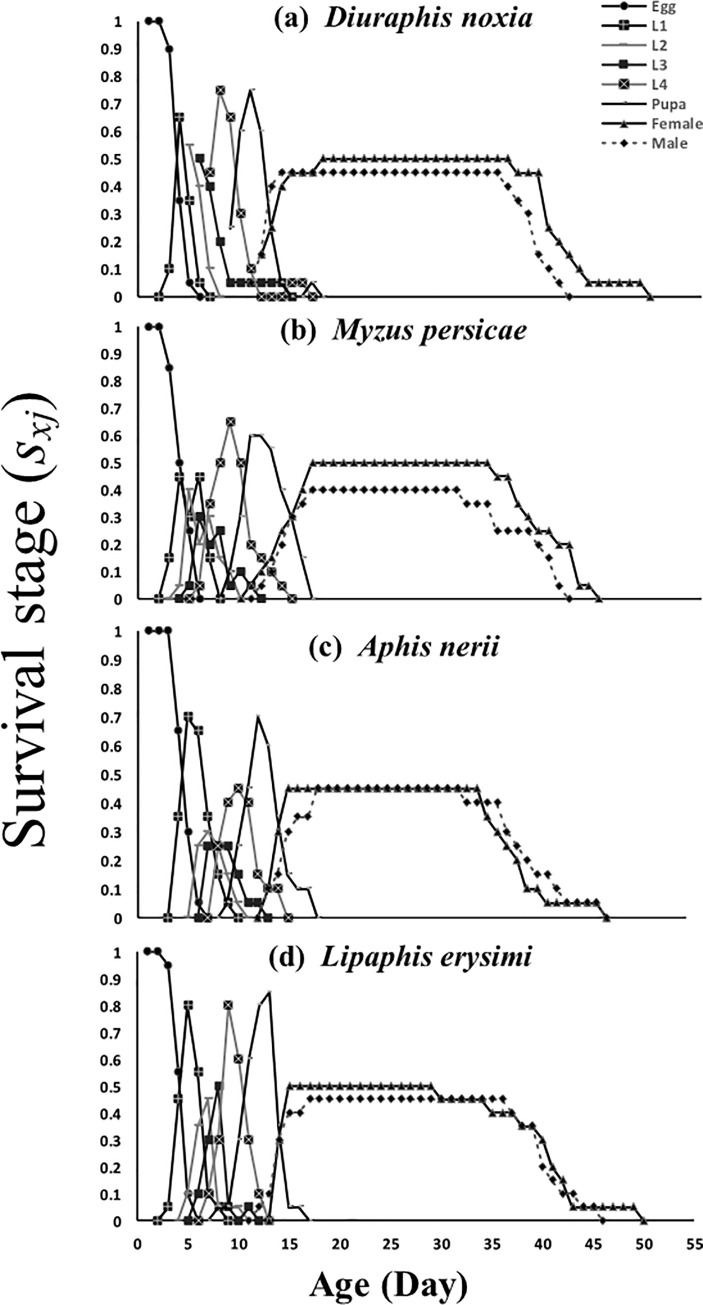
Age-stage–specific survival rate (*s*_*xj*_) of *C*. *sexmaculata* fed on four aphid species.

The maximum survival probability *S*_*xj*_*’s* of eggs, first, third and fourth instars as well as pupae of *C*. *sexmaculata* was 0.95, 0.80, 0.5 0.8, and 0.85, respectively, when *M*. *persicae* was used as food (Fig 1). However, maximum *S*_*xj*_ of second instars was 0.55 on *D*. *noxia*. The lowest survival probabilities of eggs, second, third and fourth instars of *C*. *sexmaculata* with respective values of 0.65, 0.3, 0.25 and 0.45 occurred in connection with *A*. *nerii* as food. The lowest *S*_*xj*_ of 0.45 and 0.65 for first instars and pupae, respectively, occurred with *L*. *erysimi* as food. Survival probabilities of males and females were similar in connection with all tested aphid species. (Fig 1)

**Fig 2:**
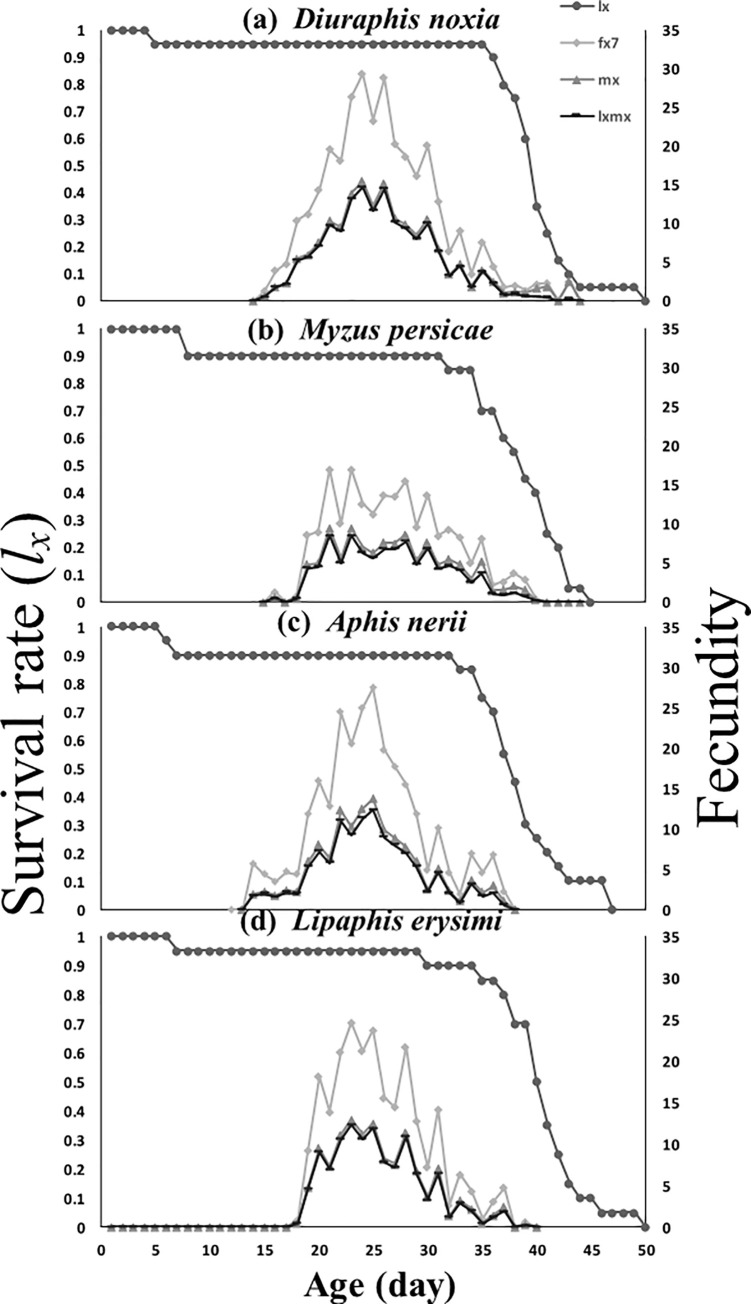
Age-specific survival rate (*l*_*x*_), age-stage–specific fecundity (*f*_*xj*_), age-specific fecundity (*m*_*x*_), and age-specific maternity (*l*_*x*_*m*_*x*_) of *C*. *sexmaculata* fed on four aphid species.

*Cheilomenes sexmaculata* evinced a higher survival rate on both *M*. *periscae* and *D*. *noxia* than *A*. *nerii* and *L*. *erysimi* as food (Fig 2). The age-stage-specific female fecundity (*f*_*x7*_) of *C*. *sexmaculata* was maximal on *D*. *noxia* (29.4 eggs at age of 23 days), but minimal on *L*. *erysimi* (15.5 eggs at the age of 27 days). Similarly, age-specific fecundity (*m*_x_) was maximal on *D*. *noxia*, but lowest on *L*. *erysimi*. Age-specific net maternity (*l*_*x*_*m*_*x*_) was highest on *D*. *noxia*. It was, however, minimal on *M*. *persicae* (Fig 2).

**Fig 3:**
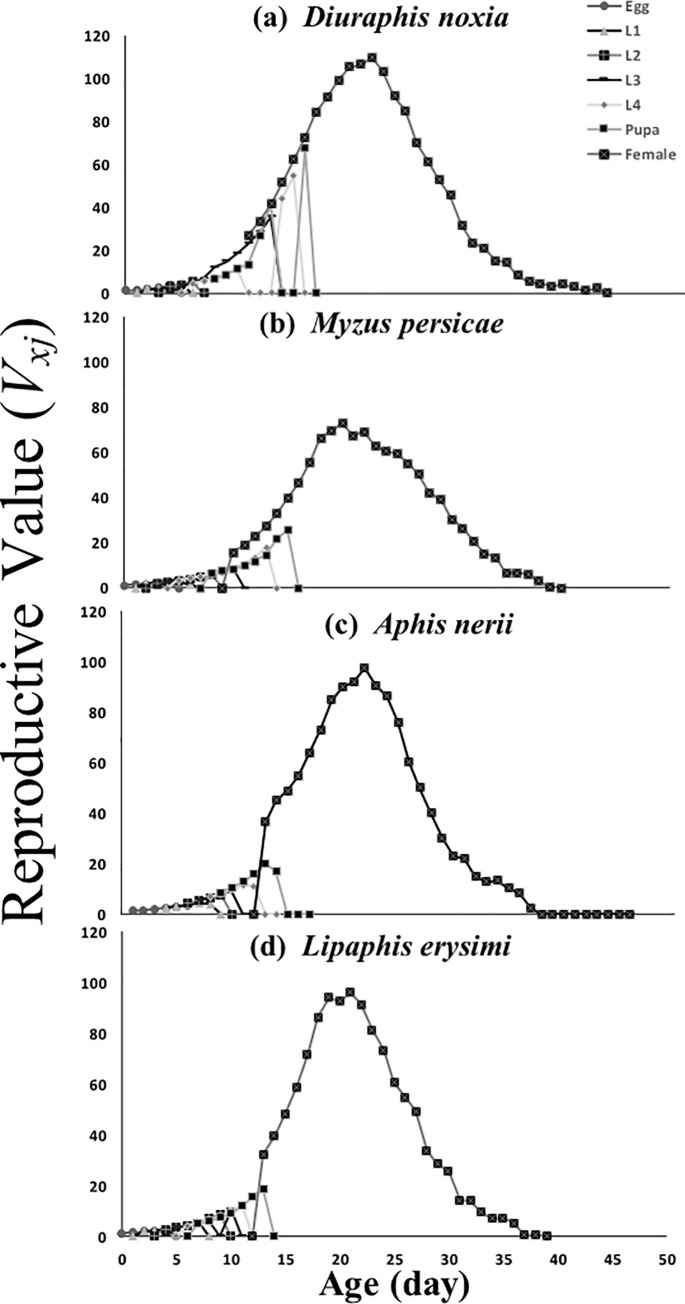
Age-stage–specific reproductive rate (*v*_*xj*_) of *C*. *sexmaculata* fed on four aphid species.

Fig 3 shows that the value of age-stage–specific reproductive rates (*v*_*xj*_) was highest in the case of *D*. *noxia* (110) at the age of 22 days compared with *A*. *nerii*, *L*. *erysimi* and *M*. *persicae* (98 at 21days, 96 at 22 days, and 73 at 20 days, respectively).

**Fig 4:**
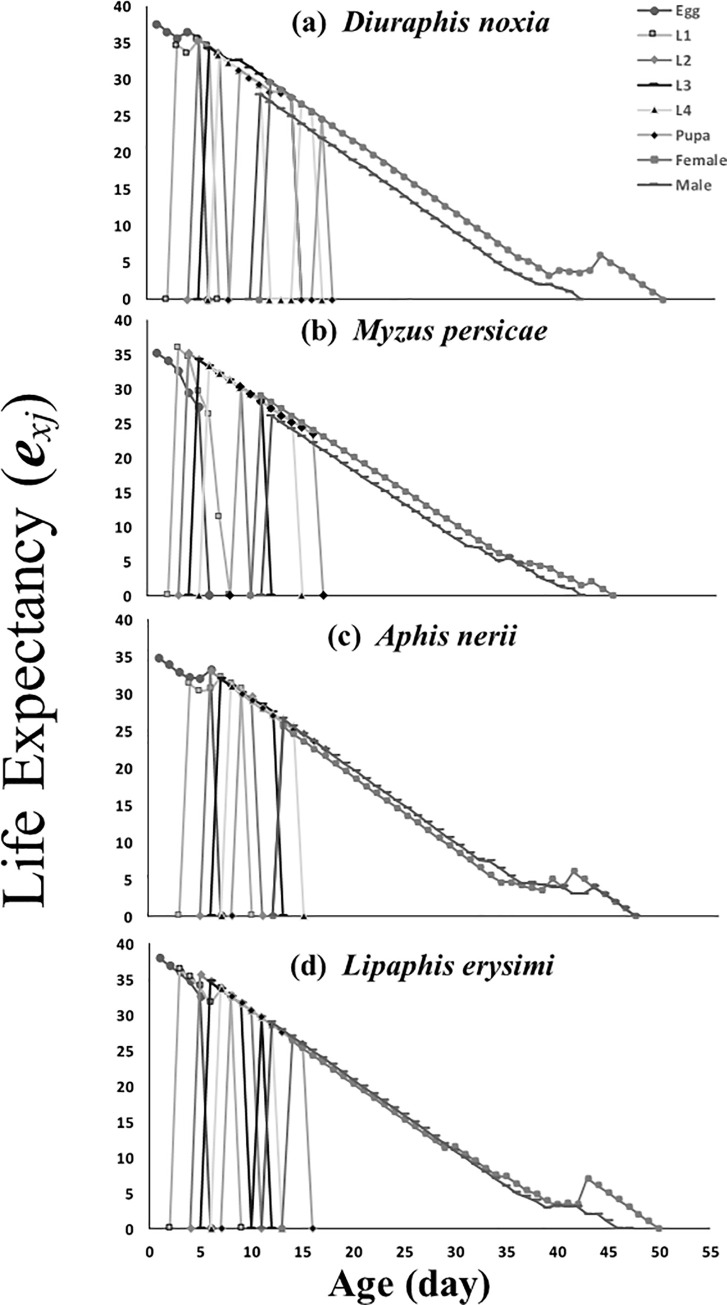
Age-stage–specific life expectancy (*e*_*xj*_) of *C*. *sexmaculata* fed on four aphid species.

Life expectancy (*e*_*xj*_) in females is higher in the case of *D*. *noxia* and *L*. *erysimi* diets than when *M*. *persicae* and *A*. *nerii* were given. Freshly hatched eggs of *C*. *sexmaculata* are estimated to live for 35, 35, 34.5 and 32.5 days on *M*. *persicae*, *L*. *erysimi*, *D*. *noxia* and *A*. *nerii*, respectively (Fig 4).

Prey consumption of *C*. *sexmaculata* significantly (*P*<0.05) varied against aphid species in all instars and adults (Table 3). To complete total larval duration, *C*. *sexmaculata* consumed mostly (*F* = 24.4; F_3,70_ and *P* = <0.0001) nymphs of *M*. *persicae* (87.42) followed by *A*. *nerii* (65.61), *L*. *erysimi* (63.83) and *D*. *noxia* (44.32). Similarly, adult pairs consumed more nymphs of *M*. *persicae* than any other aphid species (*F* = 24.5; F_3,35_ and *P* = <0.0001).

**Table 3:** Mean number of preys (different aphid species) ± SE consumed by predatory stages of *C*. *sexmaculata*.

Predator Stage	Aphid Species				Statistical parameters		
	*Aphids nerii*	*Myzus persicae*	*Diuraphis noxia*	*Lipaphis erysimi*	*F*. *value*	*df*, Edf*	*P value*
First Instar	8.50a±0.96	8.50a±1.55	1.95b±0.36	5.75c±0.63	16.2	3,76	< .0001
Second Instar	11.83a±1.49	12.21a±0.92	4.84c±0.55	8.78b±1.14	10.5	3,70	< .0001
Third Instar	17.50a±1.01	19.11a±1.70	9.11b±1.01	13.22b±2.18	8.58	3,70	< .0001
Fourth Instar	27.83b±2.45	46.37a±4.40	28.63b±2.30	35.33b±2.42	8.02	3,70	< .0001
Total larval duration	65.61b±3.43	87.42a±4.9	44.32c±2.76	63.83b±2.17	24.4	3,70	< .0001
Adults (male & female)	1630b±87.13	2482.6a±139.38	1761.0b±22.72	1610.5b±28.13	24.5	3,35	< .0001

Note: Mean followed by different letters in the same row are significantly different (*P* = 0.05)

* *df* = Degree of freedom (N-1): *Edf* = Error degree of freedom

## Discussion

This study was carried out to understand the effects that different aphid species have on the development, fecundity, and survival rate of *C*. *sexmaculata*. The results showed that the quality and availability of prey affects the development of *C*. *sexmaculata*. These results are in agreement with those of Moghaddam et al., [27] who reported that the quality and nature of the prey affected the development, fecundity and survival rate of a predator. Low quality and insufficient quantity of prey reduce the development of the predator, whereas good quality and sufficient quantity of prey increase the development of the predator [28]. Adults of *C*. *sexmaculata* feed more on aphids as compared to larval stage. This trend is also evident in *Harmonia sedecimnotata* (F.) [29, 30].

The results show considerable amount of variation among the immature stages of *C*. *sexmaculata* was recorded when fed on different aphids. First and fourth instars development was longer on *M*. *persicae* and *L*. *erysimi*, respectively. Our first instars observations are at variance with an earlier report (37) that showed a longer development of *L*. *erysimi*. This discrepancy highlights that each larval stage has a different ability to deal with plant metabolites found in their hosts. Therefore, the selection of stages of *C*. *sexmaculata* is important when put to use in the biological control program of certain target aphids. The results also showed different longevity response of males and females to the tested aphid species. Feeding on *L*. *erysimi* reduced substantially the male’s longevity, but the females longevity was same on all tested aphids. These results did not correlate with earlier studies [22, 31] in which *Coccinella septempunctata* males and females exhibited maximum longevity on *L*. *erysimi* as compared with *M*. *persicae*. This potential difference may be due to different beetle species and adults of *C*. *sexmaculata* being less affected by sequestered compounds of canola. Apparently, the females of *C*. *septempunctata* have a greater ability to detoxify metabolites present in *L*. *erysimi* than the males. Thus, females *C*. *septempunctata* individuals can be considered more promising than males as agents to control aphids, especially when *L*. *erysimi* is the main pest.

In comparison with previous studies (24, 37) the survival of *C*. *septempunctata* was surprisingly higher on *L*. *erysimi* when compared with *M*. *persicae*, whereas survival of *C*. *sexmaculata* was higher on *M*. *persicae*. This demonstrates that *C*. *sexmaculata* possesses a greater tolerance to plant metabolites than *C*. *septempunctata*. In the current study, the highest and lowest fecundity was recorded on *D*. *noxia* and *M*. *persicae*, respectively. These results are contrary to the study carried out on *C*. *septempunctata*, in which the maximum fecundity was reported on *M*. *persicae* [22, 32]. A relation exists between predator longevity and fecundity, but long longevity does not necessarily mean maximum fecundity, because the quality of the host affects both longevity and fecundity of the predator [32, 33]. All larval instars and adults of *C*. *sexmaculata* consumed nymphs of *M*. *periscae* the most. This result agrees with previous studies (37) in which the predator also consumed more *M*. *persicae*. Consumption by *C*. *sexmaculata* larvae of *A*. *nerii*, *L*. *erysimi* and *D*. *noxia* was ranked 2^nd^, 3^rd,^ and 4^th^. The most ideal host for the rearing of *C*. *sexmaculata* is *D*. *noxia* as the beetles would require fewer aphids to complete their life cycle as compared to other three aphid species. The consumption rate of *C*. *sexmaculata* shows its potential as a biological control agent against aphids, especially on *A*. *nerii* and *L*. *erysismi*, aphid species known to contain toxic plant metabolites obtained from their host plants (43).The findings of the current study revealed that the *R₀*, *λ*, *r*, *T* and *GRR* parameters are higher on *M*. *persicae* than *L*. *erysimi* in agreement with a study similar to study conducted on *C*. *septempunctata* [22, 34, 35] which concludes that *M*. *persicae* is most suitable host as compared to *L*. *erysimi*

Problems were associated with the traditional life table, i.e. considering the female population, but neglecting male populations and stage differentiation between individuals and sexes (). We used age stage, a two-sex life table to assess the difference between age-specific survival rate and age-specific fecundity, which also considered male survival and stage differentiation between individuals. The difficulties and errors associated with the female age-specific life table have briefly been addressed by [17, 36].

Our results show that age-specific mean fecundity was more on *M*. *persicae* as compared to *L*. *erysimi*, 24.6 eggs, which is in accordance with the study conducted on *C*. *septempunctata* [22, 37, 38]. The nutritional value and quality of prey species affected the predator’s fecundity [39, 40]. The life expectancy is such that an adult should be expected to live at age *x* and stage *j*. The results of this study show that life expectancy becomes reduced with the age of an adult. These results resemble those of the study conducted on *C*. *septempunctata*, namely that the adult’s life expectancy is reduced with increasing age. Under laboratory conditions it is clear that without additional stressors an adult’s life expectancy will gradually decrease with age [22, 41, 42]. The life expectancies of same-age individuals, however, may change due to differences of biological and environmental conditions in the life stages of the individuals [17].

## Conclusion

We conclude that aphid specificity affects the development, survival, and reproduction of *C*. *sexmaculata*. This study uncovers that *C*. *sexmaculata* can successfully complete its life cycle on *D*. *noxia*, *A*. *nerii* and *L*. *erysimi* and *M*. *persicae*. Life table parameters showed that *D*. *noxia* is most suitable host for *C*. *sexmaculata*. This somewhat surprising result should encourage biocontrol practitioners to use this beetle to control aphids which carry toxin from their hosts. This study also encourages the potential use of *C*. *sexmaculata* in combination with other predators, e.g. *C*. *septempunctata* and *Chrysoperla carnea* (Neuroptera: Chrysopidae), who failed to feed on certain preys (like *L*. *erysimi*), which contained plant metabolites in their bodies from their host plants [43, 44]. *Brassica* strips are commonly employed in the wheat ecosystem for propagation of natural enemies [45]. This study supports the potential inoculative release of *C*. *sexmaculata* on *Brassica* strips for propagation to control *D*. *noxia* in wheat crops, but this needs to be tested. Our findings show the potential of *C*. *sexmaculata* as a biocontrol agent not only in cultivated crops but also in connection with ornamental plants based on the specie’s life table parameters with various aphid species as prey involved. However, it is important to consider the most suitable stage of *C*. *sexmaculata* to use against targeted aphid species. The age-stage two-sex life table explored information on the efficacy and potential use of the *C*. *sexmaculata* in connection with the biological control of aphids. Future studies should provide additional details on the aphid species preferred by *C*. *sexmaculata* and further studies focusing on field applications of *C*. *sexmaculata* for the management of aphids are required.
